# Supplementary figures and images for: Attack of the dark clones the genetics of reproductive and color traits of South African honey bees (Apis mellifera spp.)
Source: PLoS One. 2021 Dec 14;16(12):e0260833. doi: 10.1371/journal.pone.0260833 (PMC8670704; doi:10.1371/journal.pone.0260833)

S2 File. Candidate variant distribution of alleles per subspecies

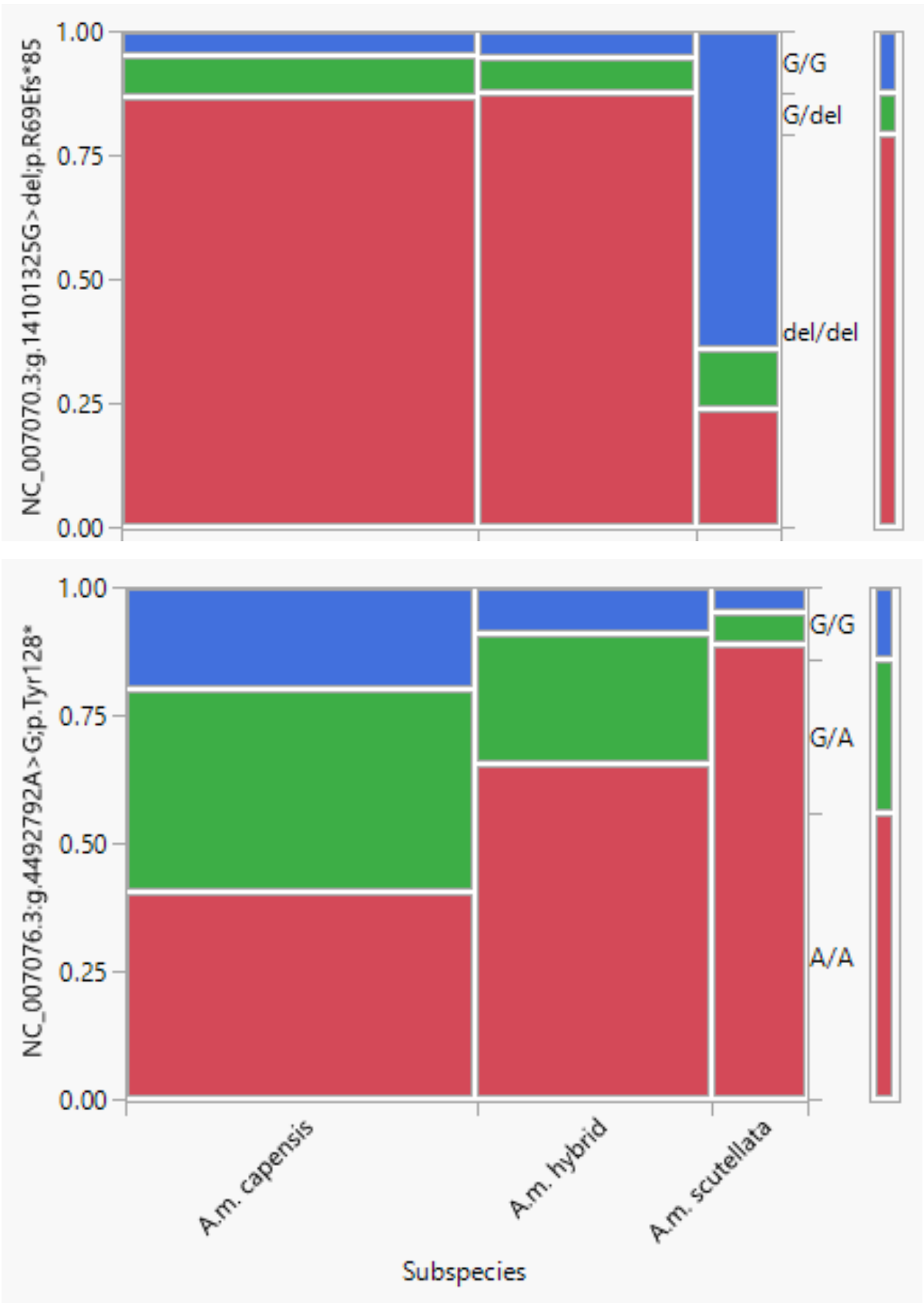

Supplement: S2 File — (PDF) [file pone.0260833.s002.pdf]
