# Supplementary material for: Attack of the dark clones the genetics of reproductive and color traits of South African honey bees (Apis mellifera spp.)
Source: PLoS One. 2021 Dec 14;16(12):e0260833. doi: 10.1371/journal.pone.0260833 (PMC8670704; doi:10.1371/journal.pone.0260833)
Supplement: S1 Table — Hybrid = A cross between A.m. scutellata and A.m. capensis. NA = bee samples from that location were not included in the respective analysis. The “Combined Probable ID” is inferred from the most common identification (ID) made across the four referenced studies and it parallels the identifications assigned using SNPs. (PDF) [file pone.0260833.s003.pdf]

S1 Table. Sample *Apis mellifera* subspecies assignment per source information. Hybrid = A cross between *A.m. scutellata* and *A.m. capensis*. NA = bee samples from that location were not included in the respective analysis. The "Combined Probable ID" is inferred from the most common identification (ID) made across the four referenced studies and it parallels the identifications assigned using SNPs.

**Geographic region within South Africa**	**Morphometrics** [26]	**SNPs** [18]	**Microsatellites** [54]	**Mitogenome** [53]	**Combined Probable ID**
Beaufort West – BW	hybrid	hybrid	hybrid	*A.m. capensis*	hybrid
Bloemfontein – BL	*A.m. scutellata*	*A.m. scutellata*	*A.m. scutellata*	*A.m. capensis*	*A.m. scutellata*
Bredasdorp – BD	*A.m. capensis*	*A.m. capensis*	*A.m. capensis*	*A.m. capensis*	*A.m. capensis*
Cape Town – CT	*A.m. capensis*	*A.m. capensis*	*A.m. capensis*	*A.m. capensis*	*A.m. capensis*
Citrusdaal – CD	*A.m. capensis*	*A.m. capensis*	hybrid	*A.m. scutellata*	*A. m. capensis*
East London – EL	hybrid	hybrid	hybrid	NA	hybrid
George – GE	*A.m. capensis*	*A.m. capensis*	hybrid	*A.m. capensis*	*A.m. capensis*
Graaff-Reinet – GR	hybrid	hybrid	hybrid	NA	hybrid
Grahamstown – GT	hybrid	hybrid	hybrid	*A.m. capensis*	hybrid
Klawer – KL	hybrid	hybrid	hybrid	*A.m. scutellata*	hybrid
Knysna – KN	*A.m. capensis*	hybrid	hybrid	*A.m. capensis*	hybrid
Kroonstad – KR	*A.m. scutellata*	*A.m. scutellata*	hybrid	*A.m. scutellata*	*A.m. scutellata*
Laingsburg – LB	*A.m. capensis*	*A.m. capensis*	hybrid	*A.m. scutellata*	*A. m. capensis*
Langebaan – LA	*A.m. capensis*	*A.m. capensis*	hybrid	*A.m. capensis*	*A.m. capensis*
Moorreesburg – MB	*A.m. capensis*	*A.m. capensis*	*A.m. capensis*	*A.m. capensis*	*A.m. capensis*
Modderfontein – MF	*A.m. capensis*	hybrid	*A.m. scutellata*	*A.m. capensis*	hybrid
Oudtshoorn – OD	*A.m. capensis*	*A.m. capensis*	*A.m. capensis*	NA	*A.m. capensis*
Plettenburg Bay - PB	*A.m. capensis*	hybrid	*A.m. capensis*	*A.m. capensis*	hybrid
Port Elizabeth – PE	*A.m. capensis*	hybrid	*A.m. capensis*	*A.m. capensis*	hybrid
Pretoria – PT	*A.m. scutellata*	*A.m. scutellata*	*A.m. scutellata*	*A.m. scutellata*	*A.m. scutellata*
Riversdale – RD	*A.m. capensis*	*A.m. capensis*	*A.m. capensis*	*A.m. capensis*	*A.m. capensis*
Saint Francis - SF	*A.m. capensis*	hybrid	hybrid	*A.m. capensis*	hybrid
Springbok – SP	*A.m. scutellata*	*A.m. scutellata*	*A.m. scutellata*	*A.m. scutellata*	*A.m. scutellata*
Stellenbosch – ST	*A.m. capensis*	*A.m. capensis*	*A.m. capensis*	*A.m. scutellata*	*A.m. capensis*
Swellendam -SW	*A.m. capensis*	*A.m. capensis*	*A.m. scutellata*	*A.m. capensis*	*A.m. capensis*
Touwsrivier – TR	*A.m. capensis*	*A.m. capensis*	*A.m. capensis*	NA	*A.m. capensis*
Upington - UP	*A.m. scutellata*	*A.m. scutellata*	*A.m. scutellata*	*A.m. scutellata*	*A.m. scutellata*
Vryburg – VR	*A.m. scutellata*	NA	*A.m. scutellata*	*A.m. scutellata*	*A.m. scutellata*
Worcester - WD	hybrid	*A.m. capensis*	*A.m. scutellata*	*A.m. capensis*	*A. m. capensis*
